# Serum fatty acid binding protein 5 (FABP5) as a potential biomarker of inflammation in psoriasis

**DOI:** 10.1007/s11033-021-06461-3

**Published:** 2021-06-15

**Authors:** Dorota Kozłowska, Hanna Myśliwiec, Ewa Harasim-Symbor, Anna Justyna Milewska, Adrian Chabowski, Iwona Flisiak

**Affiliations:** 1 Department of Dermatology and Venereology, Medical University of Bialystok, Żurawia str. 14, 15-540 Białystok, Poland; 2 Department of Physiology, Medical University of Bialystok, Białystok, Poland; 3 Department of Statistics and Medical Informatics, Medical University of Bialystok, Bialystok, Poland

**Keywords:** Psoriasis, Epidermal fatty acid binding protein, Fatty acid binding protein 5, Narrowband—ultraviolet B, Metabolic syndrome, Lipid disturbances

## Abstract

Fatty acid binding protein 5 (FABP5) is elevated in psoriatic keratinocytes and could be involved in systemic metabolic disturbances in psoriasis. The aim of the study was to evaluate serum FABP5 in obese and non-obese psoriatic patients, to assess the relationship between FABP5 and the duration, severity of the disease, inflammatory and metabolic markers and influence of treatment with narrowband—ultraviolet B (NB-UVB). Seventy-four patients (30 treated with NB-UVB) with psoriasis were enrolled in the study. The serum concentrations of FABP5 were measured using Human FABP5 Enzyme-Linked Immunosorbent Assay kit. Serum fatty acids were measured by gas–liquid chromatography. Serum FABP5 levels in psoriatic patients were higher versus control group (*P* < 0.001). FABP5 in patients with PASI > 20 was higher compared to the mild group (PASI < 10) (*P* < 0.001) and serum FABP5 correlated positively with PASI score (r = 0.41, *P* < 0.001). There was also positive correlation between FABP5 and basic inflammation indices. Decrease of PASI after NB-UVB treatment (*P* < 0.001) was observed and accompanied by decrease of the serum FABP5 (*P* = 0.007). FABP5 is a potential marker of psoriasis, its severity and clinical outcome after therapy with NB-UVB. FABP5 may reflect metabolic disturbances in psoriatic patients.

## Introduction

Psoriasis is an immune-mediated, chronic inflammatory skin disease of complex patomechanism. Several studies have shown that psoriasis is associated with systemic disorders specially metabolic syndrome (MS), which is defined as a constellation of insulin resistance, obesity, hyperlipidemia and hypertension [1, 2] Psoriatic patients are also predisposed to other comorbidities like: inflammatory bowel diseases, cardiovascular diseases and non-alcoholic fatty liver disease (NAFLD) [3–5]. It has been established that the release of inflammatory molecules and cytokines play an important role in metabolic disturbances and atherosclerosis in the course of psoriasis [6]. On the other hand, there are some evidence that pre-existing obesity or MS increase the risk of development of psoriasis [7]. The possible explanation of this bilateral interinfluence is the chronic, systemic metabolic inflammatory state, termed metaflammation [8]. Despite of advancement in medical science and new methods of psoriasis treatment, mortality rate in psoriatic patients is still 20% higher than in the general population [9]. Although abnormalities in lipid metabolism play an important role in the pathogenesis of psoriasis and its comorbidities, the exact underlying mechanism is complex and still unclear.

In recent years, a number of proteins facilitating fatty acid transport, including fatty acid binding protein (FABP) have been identified. These proteins, do not only buffer lipids, but also are crucial mediators of metabolic and other biological activities [10]. In previous studies increase of serum FABP2, FABP4 in the psoriatic patients have been found [11, 12]. Fatty acid binding protein 5 (FABP5, or Epidermal Fatty Acid Binding Protein, E-FABP) is a lipid carrier, originally discovered in human epidermis. FABP5 is predominantly expressed on keratinocytes, but also abundantly in adipose tissue (adipocytes and macrophages), in thymus and tongue epithelia [13, 14]. Psoriatic post mitotic keratinocytes overexpress FABP5, as compared to the keratinocytes, in normal epidermis. FABP5 induces differentiation in normal human keratinocytes and modulates the differentiation process in psoriatic keratinocytes in vitro [14]. Although the role of FABP5 in the keratinocytes in psoriatic epidermis has been already elucidated, the role of circulating FABP5 has not been studied extensively in this population. Other studies revealed that FABP5 may represent mediators of and biomarkers for metabolic and cardiovascular disease in type 2 diabetes mellitus, thus it could play an important role and contribute to the development of cardiometabolic comorbidities in psoriasis [15, 16].

The aim of the present study was to evaluate serum concentration of FABP5 in psoriatic patients and to assess the relationship between FABP5 and the duration and the severity of the disease as well as the inflammatory and metabolic markers. Additionally, the influence of narrowband—ultraviolet B (NB-UVB) treatment of psoriasis on FABP5 serum concentration have been investigated.

## Methods

### Subjects and examination

The study was conducted on 74 patients (25 women and 49 men) with exacerbated plaque psoriasis aged 19 to 79 years (mean 50.7 ± 14.5 years) with active plaque-type psoriasis and 30 sex- and age-matched healthy controls. None of the patients or controls were under any dietary restriction. All patients were white people and had skin phototype II and III. They did not use phototherapy or any other systemic therapy of psoriasis during the previous 3 months. Patients suffering type II diabetes, liver disease, using lipid-lowering drugs, and systemic therapy for psoriasis were excluded from the study. The duration of psoriasis varied from 1 to 58 months (mean 18.7 months). The severity of psoriasis was assessed using Psoriasis Area and Severity Index (PASI) [17]. All subjects gave their informed consent for inclusion before they participated in the study. The study was conducted in accordance with the Declaration of Helsinki, and the protocol was approved by the Bioethical Committee of the Medical University of Bialystok, Poland (No: R-I-002/457/2016).

### NB-UVB treatment

The group of 30 patients underwent NB-UVB treatment. Blood samples were collected before and after phototherapy. The treatment schedule comprised irradiations three times a week, starting from the dose 0.020 J/cm2 for the II phototype and 0.024 J/cm2 for the III phototype. The dose was increased by approximately 10%-20% per week, depending on skin type, tolerance and clinical response to the therapy. In case of signs of burning, the dose was diminished or the treatment was stopped for two–three days. TL-01 lamps (Cosmedico Medizintechnik GmbH, Stuttgart, Germany) were used as a source of irradiation (wavelength of 311–313 nm). Some patients completed 20 lamp treatment, some finished irradiations earlier for different family and social reasons. The patients underwent 14.9 ± 4.5 (min 6, max 20) irradiations and their cumulative dose was 0.84 ± 0.43 J/cm^2^ (min 0.2, max 2.28). During the whole period of irradiations the patients were asked to apply only topical emollients on the skin. PASI, the number of irradiations and the total dose of irradiations at the end of the treatment were calculated.

### Blood collection and analyses

Blood was taken after overnight fast before starting the treatment. In the group of patients treated with NB-UVB the blood was collected twice: before and after phototherapy, after overnight fast. After centrifugation the samples have been stored at − 80 °C until analysis.

The serum concentrations of FABP5 were measured using commercially available Human FABP5 enzyme-linked immunosorbent assay (ELISA) kit (BioVendor, Karasek, Brno, Czech Republic). All assay procedures were conducted following the manufacturer’s instruction. Four fold dilution of each sample was required before analysis. The limit of detection for FABP5 was 0.066 ng/mL, and the standard curve range was between 1–40 ng/mL. Intra- and inter-assay coefficients of variations were equal to 5.8% and 6.1%, respectively. At the end of the procedure the intensity of colored product was measured in a hybrid multi-mode microplate reader (Synergy H1™, Biotek Instruments, Winooski, Vermont, USA) at 450 nm wavelength and the absorbance of the samples was plotted on standard curve. The biochemical analysis including C-reactive protein (CRP), serum fasting blood glucose (FBG), total cholesterol, high-density lipoprotein cholesterol (HDL-C), low-density lipoprotein cholesterol (LDL-C), triglycerides (TG), aspartate aminotransferase (AST), alanine aminotransferase (ALT), bilirubin were performed in the Central Laboratory of the University Hospital Center.

Total serum fatty acids content and composition was measured according to a method by Glaser et al. [18]. Briefly, 100µL of serum was incubated at 85 °C for 45 min in 1.5 mL of 3 N HCl in methanol containing 2 g/l BHT (2,6-di-tert-butyl-p-cresol, antioxidant). Prior to incubation, 100 μl of internal standard mixture (heptadecanoic acid, cholesteryl-heptadecanoate, triheptadecanoate, diheptadecanoate and diheptadecanoyl-phosphatidylcholine; 0.2/2/1.5/0.2/2 per weight, 10 μg of C17:0 total, in chloroform/methanol 2:1) was added to account for methylation and extraction losses. After cooling to room temperature, fatty acids methyl esters were extracted with 0.5 mL hexane, 30 s of vortexing and centrifugation (3000 g for 5 min). A volume of upper organic phase was transferred glass vials and 1µL of sample was analyzed by gas–liquid chromatography using a Hewlett-Packard 5890 Series II gas chromatograph, a Agilent J&W CP-Sil 88 capillary column (50 m × 0.25 mm I.D.) and flame-ionization detector. The oven temperature was programmed from 130 °C to 220 °C at 5 °C/min and held at 220 °C for 32 min. Argon was used as carrier gas. The following fatty acid species were identified and quantified according to respective retention times of synthetic standards: myristic (14:0), palmitic (16:0), palmitoleic (16:1n-7), stearic (18:0), oleic (18:1n-9), linoleic (18:2n-6), α-linolenic (18:3n-3), arachidic (20:0), arachidonic (20:4n-6), eicosapentaenoic (20:5n-3), behenic (22:0), docosahexaenoic (22:6n-3), lignoceric (24:0) and nervonic (24:1n-9) acids. The FA were additionally grouped according to their biologic properties. The percentage of SFA (myristic acid, palmitic acid, stearic acid, arachidic acid, behenic acid, lignoceric acid) were measured and calculated. Unsaturated fatty acids (UFA) were divided into monounsaturated fatty acids (MUFA) (palmitoleic acid, oleic acid, nervonic acid), and polyunsaturated fatty acids (PUFA) (n-3 PUFA: α-linolenic acid, eicosapentaenoic acid (EPA), docosahexaenoic acid (DHA) and n-6 PUFAs: linoleic acid, arachidonic acid).

### Statistical analysis

Descriptive statistics were used to show sociodemographic and biochemistry characteristics of the study groups. According to the Shapiro–Wilk test the examined variables did not have normal distribution. Data were presented as median and quartiles. In the statistical analysis Mann–Whitney tests and Kruskal–Wallis tests were used. The correlations between the serum FABP5 and metabolic and clinical variables were determined by Spearman’s rank correlation analysis. The difference was statistically significant at *P* < 0.05.All analysis was performed using Statistica software (version 12.0).

## Results

Seventy four patients (25 women and 49 men) with exacerbated plaque type psoriasis patients, aged 19 to 79 years (mean 50.7 ± 14.5 years). The control group consisted of 30 age- and sex-matched healthy controls. The clinical and demographic data of the patients have been shown in Table 1. The median severity of the disease measured by PASI was 9.05 (from 1.4 to 38.3). From the first diagnosis psoriasis lasted from 1 to 58 months (mean 18.7 months). In the study group 43 patients (58.1%) had mild disease (PASI < 10), 21 (28.4%) had moderate disease (PASI 10–20) and 10 (13.5%) were diagnosed with severe disease (PASI > 20).

**Table 1:** Clinical and laboratory characteristics of the psoriatic patients and the controls

Parameter	Psoriasis (n = 74) Median (Q1,Q3)	Controls (n = 30) Median (Q1,Q3)	p-value
Age (years)	54.0 (41.0; 59.0)	42.0 (38.0; 54.0)	NS
BMI (kg/m^2^)	27.7 (23.89;33.09)	23.81 (22.22; 26.02)	P = 0.001
Men: Women	49(66.2%):25(33.8%)	20(67%):10(33%)	–
Psoriasis duration (months)	17.0 (6.0; 30.0)	–	–
PASI score	9.05 (5.9; 13.8)	–	–
Serum FABP5 concentration	16.27 (12.42; 21.69)	7.81 (6.33; 9.26)	P < 0.001
Serum vitamin D concentration	15.43 (11.16; 21.92)	21.29 (16.38; 27.67)	NS

Data shown as median and quartiles (Q_1_—first quartile; Q_3_—third quartile) and percentage. (BMI- body mass index, PASI – psoriasis area and severity index, NS – non-significant)

**Fig. 1:**
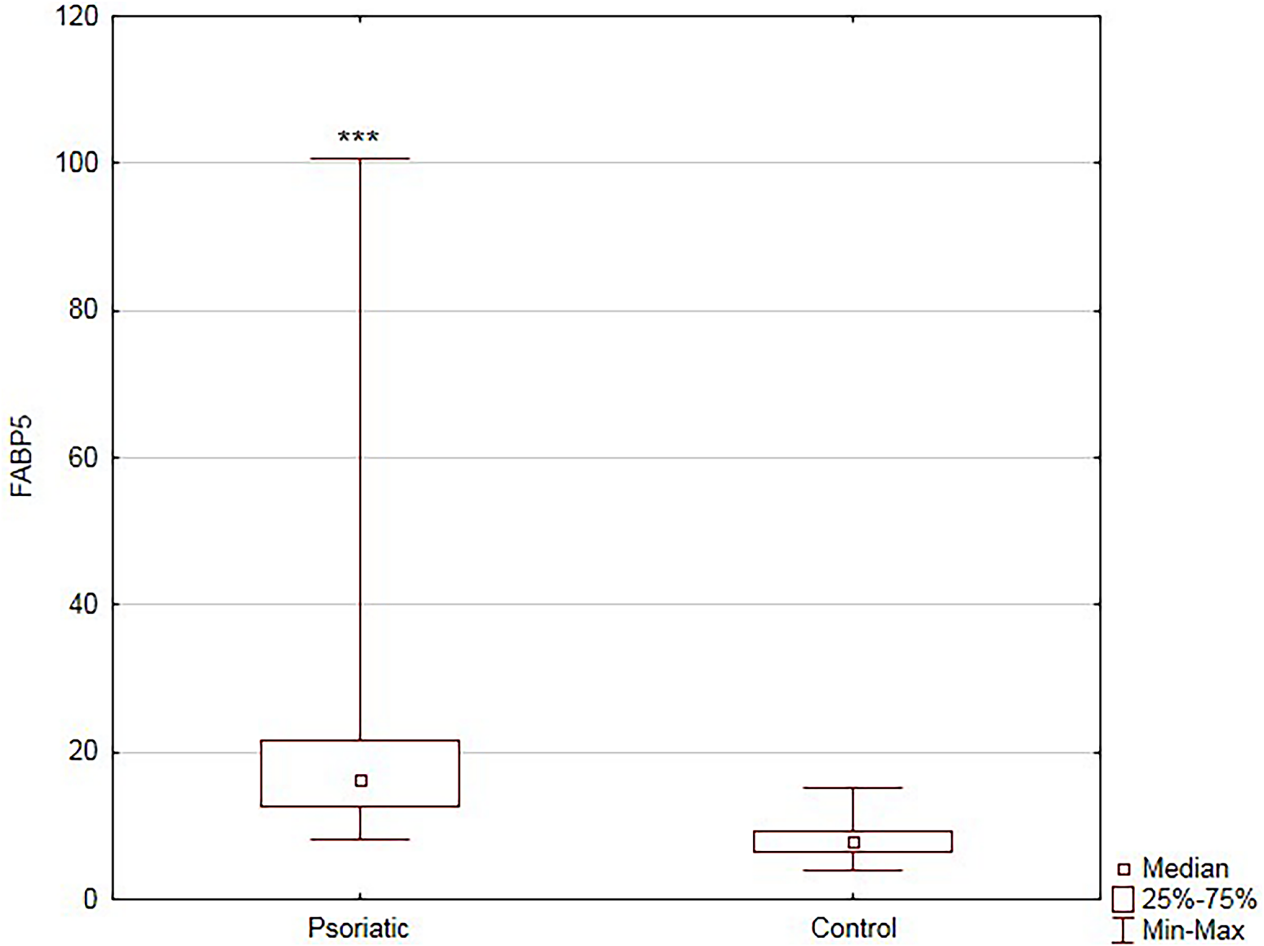
Comparison of serum fatty acid binding protein 5 (FABP5) concentration between patients and controls. Data shown as median and quartiles (Q1, Q3). Significant difference between the groups *P* < 0.001 *** (n = 74)

The median serum FABP5 concentration in the study group (16.28 ng/mL; Q1 = 12.72400, Q3 = 21.69600) was significantly higher than in the healthy controls (7.82 ng/mL; Q1 = 6.336000; Q3 = 9.2600) (*P* < 0.001) (Fig. 1). Futhermore, the positive correlation between serum FABP5 concentration and PASI score before treatment was observed in patients with psoriasis (R = 0.42; p < 0.001).

To avoid the influence of obesity on the study results, the examined group was divided to non-obese patients (n = 50, BMI < 30 kg/m^2^) and obese patients (n = 24, BMI ≥ 30 kg/m^2^). Non-obese group consists of 26 patients with normal body weight (19 ≥ BMI < 25) and 24 individuals with overweight (25 ≥ BMI < 30). There was no significant difference between BMI of normal weight and overweight psoriatic patients. Both obese and non-obese psoriatic patients had higher FABP5 than the healthy control group (*P* < 0.001 and *P* < 0.001 respectively).

**Fig. 2:**
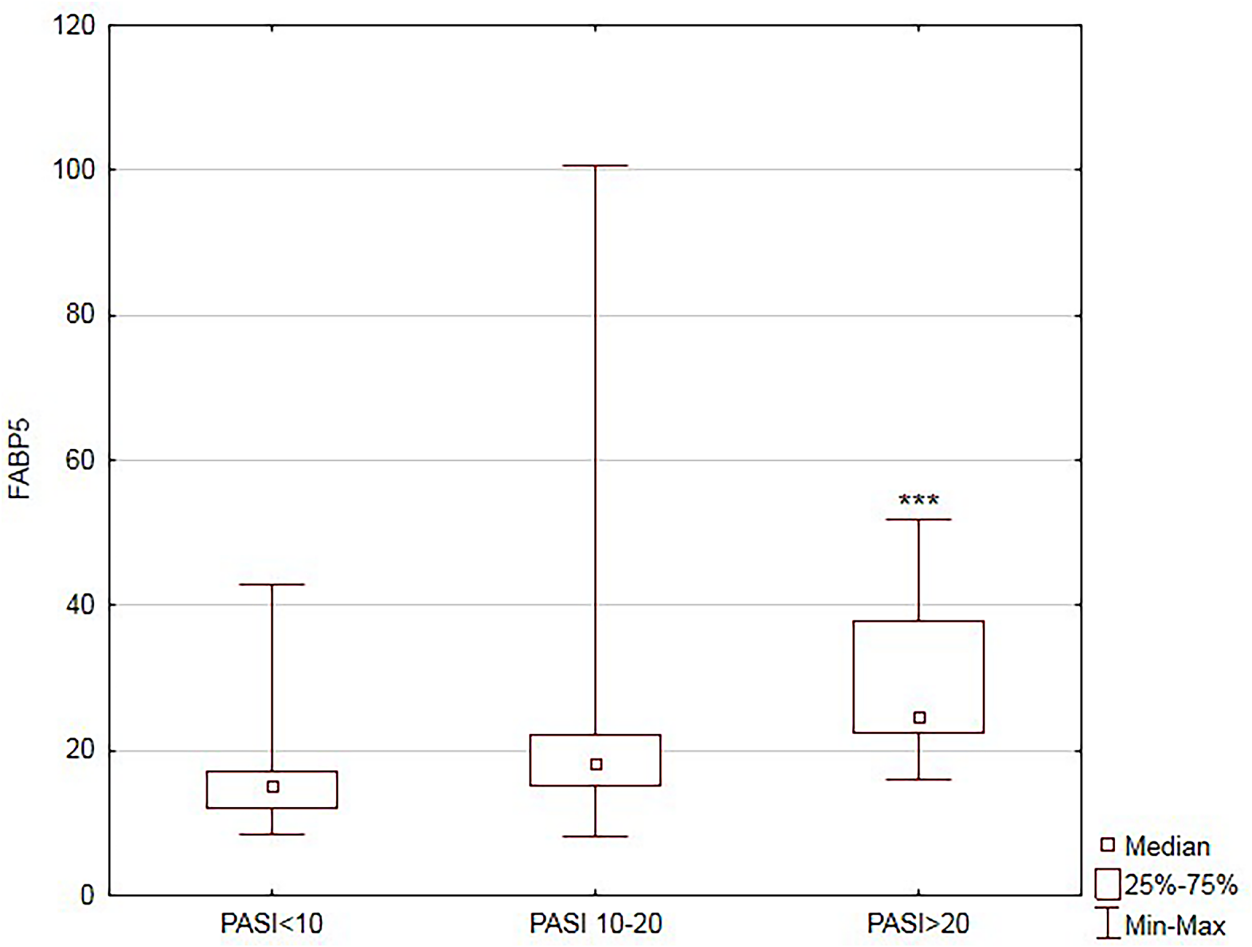
Comparison of serum fatty acid binding protein 5 (FABP5) concentrations between patients according to the severity of the disease. Data shown as median and quartiles (Q1, Q3). Significant difference between the groups *P* < 0.001*** (PASI – psoriasis area and severity index) (n = 74)

Based on psoriasis activity FABP5 concentration in patients with severe form of the disease (PASI > 20) was higher compared to the mild group (PASI < 10) (*P* < 0.001) (Fig. 2).

**Fig. 3:**
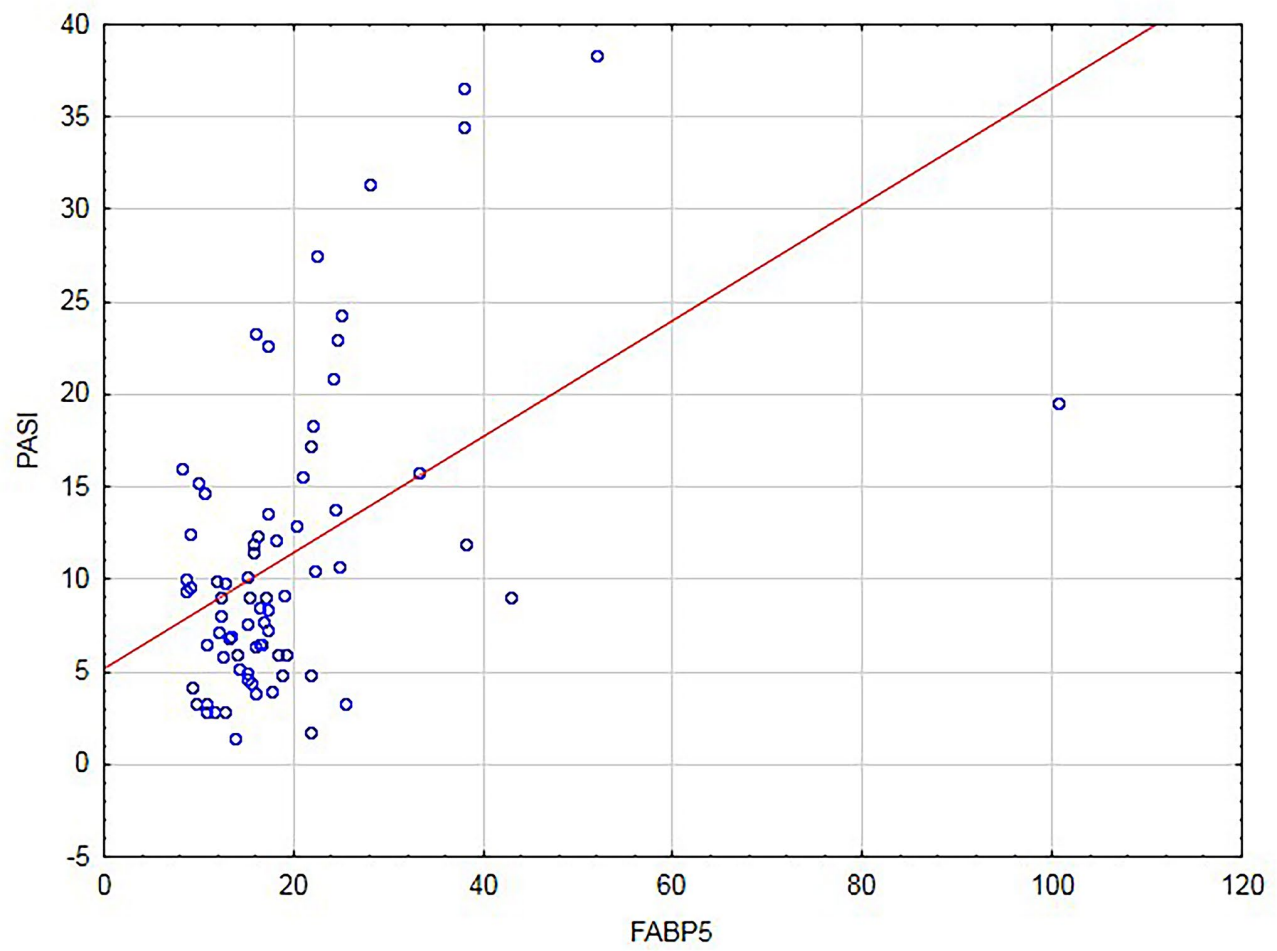
Correlation between concentrations of serum fatty acid binding protein 5 (FABP5) and PASI (psoriasis area and severity index) (n = 74) (r = 0.42, *P* < 0.001) (n = 74)

Serum FABP5 correlated positively with PASI score (r = 0.42, *P* < 0.001) in all psoriatic patients (n = 74) (Fig. 3). The similar correlation was observed in the patients group with BMI < 30 (r = 0.41, *P* = 0.003). There was no correlation between PASI score and FABP5 in obese psoriatic patients. There was no relationship between FABP5 and disease duration or age of the patients.

There was positive correlation between FABP5 and basic inflammation indices: C-reactive protein (R = 0.29, p = 0.014), white blood cell count (R = 0.50; p < 0.001) and the platelet count (R = 0.26; p = 0.033). Negative correlation between FABP5 and serum vitamin D concentration before NB-UVB treatment was found (R = -0.24; p = 0.040).

There was also some correlations between FABP5 with the serum free fatty acids—negative correlations between FABP5 and n-3 PUFA (R = -0.28; p = 0.017), arachidonic acid (R = -0.36; p = 0.002), eicozapentaenoic acid (R = − 0.28; p = 0.014) and docozaheksaenoic acid (R = − 0.27, p = 0.021). Positive correlation between FABP5 and n-6/n-3 PUFA ratio was observed (R = 0.28; p = 0.017).

After the treatment with NB-UVB the median total PASI score decreased from the basal PASI 6.4 to 2.0 (*P* < 0.001). FABP5 serum concentration decreased significantly from median 6.89 to 6.5 (*P* = 0.007) (Table 2).

**Table 2:** PASI and FABP5 serum concentration before and after treatment with NB-UVB. Data are shown as median and quartiles (first quartile—Q_1_,third quartile- Q_3_)

	Before NB-UVB treatment	After NB-UVB treatment	*P* -value
PASI	6.4 (4.4; 8.0)	2.0 (1.4; 2.7)	*P* < 0.001
FABP5 ng/mL	6.89 (5.69; 9.05)	6.51 (3.8; 8.15)	*P* = 0.007

*PASI* psoriasis area and severity index (n = 30)

## Discussion

Serum concentration of FABP5 in psoriatic patients was examined with respect to clinical and laboratory data. Significantly higher levels of FABP5 were observed in both groups of psoriatic patients (with and without obesity) compared to the healthy control subjects. The obtained results were different to results of Miyake et al. who found similar average serum FABP5 level in psoriatic patients and healthy individuals [19]. However authors discovered higher FABP5 level in skin-stripping lesions and uninvolved skin of patients than the skin of healthy individuals. There were some differences between the study group in the present study and patients of Miyake. In this study there were 74 patients with less severe disease (PASI mean 11.3) versus 31 patients (PASI mean 17.3). Studied patients did not use any topical or systemic treatment prior to the study. Patients of Miyake used topical steroids and/or vitamin D ointments. Topical treatment, specially steroids and vitamin D, may have some immunomodulatory effect and influence the results. There are several studies confirming high expression of FABP5 in psoriatic skin lesions and in uninvolved skin of psoriatic patients compared to the healthy skin [14, 19, 20]. Authors suggest that FABP5 plays an important role in keratinocyte differentiation [14]. Higher serum concentration of FABP5 in this research may reflect its epidermal level. The underlying mechanism of the association of FABP5 and psoriasis is not clear yet, but one of the possible explanation is role of the adaptive immune system, particularly of Th1 and Th17 lymphocytes, which has been regarded as prominent in the immunopathogenesis of psoriasis [21]. Interestingly, studies have shown that FABP5 can increase Th17-cell differentiation [22] and aberrant FABP5 activity may over activate immune cells leading to inflammatory autoimmune diseases [23].

The positive correlation of serum FABP5 with basic inflammation indices: C-reactive protein, white blood cell count and the platelet count in the current study, seems to confirm the notion that FABP5 follows the inflammatory process in psoriasis and may play an important role in the pathogenesis of psoriasis and, what is more, may be used as a biomarker of severity of the disease [24, 25].

High level of FABP5 can have also some metabolic implications. In the other studies serum FABP5 levels correlated positively with parameters of adiposity, adverse lipid profiles, serum insulin, adipocyte fatty acid binding proteins (A-FABP, FABP4), C-reactive protein levels and were higher in subjects with the metabolic syndrome (MS). According to Yeung et al. FABP5 is a new circulating biomarker associated with increased cardio-metabolic risk and the association of FABP5 with the MS and the carotid intima thickness is independent of FABP4 [26]. Similar, Bagheri et al. suggested that FABP4 and FABP5 may represent mediators of and biomarkers for metabolic and cardiovascular disease in type 2 diabetes mellitus [16]. Moreover, Ibarretxe et al. suggested that FABP5 was associated with increased subclinical atherosclerosis and higher FABP5 plasma levels was associated with the presence of type 2 diabetes, obesity, metabolic syndrome, or atherogenic dyslipidemia [27]. The meta-analysis of 35 studies and 1,450,188 participants revealed that psoriatic patients have higher prevalence to MS compared to general population. [2]. In psoriasis MS is diagnosed in about 31.4% of patients [28]. Subsequently, numerous studies have demonstrated that psoriasis, particularly severe disease, is associated with increased risk of cardiovascular diseases [8, 29]. Present results may indicate that elevated serum FABP5 represents another link between psoriasis MS and cardiovascular comorbidities.

On the other hand, in the last decade, modern epidemiological studies have provided strong evidence that obesity predisposes patients to the development of psoriasis and amplifies psoriatic inflammation [30, 31] The analysis of metabolic factors indicated that adiposity is a central factor in this association [32]. Zhang Y et al. reported that FABP5 expression from the high-fat diet-induced obese mice was twofold higher in keratinocytes and sixfold higher in macrophages as compared to the controls. This results indicate the high-fat diet upregulates FABP5 in the skin tissues and promotes inflammation of the skin [33]. Sometimes it is difficult to point out what has came first in the metabolic dysregulation or inflammation. On both sides, elevated serum FABP5 observed in this study may contribute to the further immunologic and metabolic dysregulation.

Additionally, there was a negative correlation of serum FABP5 with serum concentration of arachidonic (AA), eicosapentaenoic (EPA), docosahexaenoic (DHA) fatty acids and positive correlation with n-6/n-3 ratio of polyunsaturated fatty acids (PUFA). According to the literature, FABP5 has a broad range of ligands, including saturated and unsaturated fatty acids like: linoleic acid, EPA, DHA, AA and their derivatives [34]. In general, EPA and DHA are able to inhibit different mechanism of inflammation while n-6 PUFA promote production of inflammatory cytokines and T cell reactivity through production of prostaglandins and leukotrienes from AA [35]. Positive correlation of FABP5 with n-6/n-3 ratio highlights the pro-inflammatory state promoting metabolic disturbances in psoriatic patients.

Psoriasis is often accompanied by NAFLD. There was no correlation of FABP5 with liver function tests in the present study. However, such connection was reported by Ishimura in general population [36]. Present study group was free of liver diseases and the levels of transaminases were within the normal range so, based on present results, it is difficult to conclude about connection of FABP5 and NAFLD in psoriasis.

To the best of our knowledge, this is the first study evaluating influence of NB-UVB on serum concentration of FABP5 in patients with chronic plaque psoriasis. Significant decrease of serum FABP5 after the effective NB-UVB treatment was observed. There was only one study comparing an effect of different methods of treatment on FABP5 levels in skin-stripping [17]. The authors have reported decreased levels of skin FABP5 among 7 (out of 10) studied patients. Two of them were treated with adalimumab, 3 with infliximab and 2 with NB-UVB. It is difficult to compare this results with present study. Firstly, because FABP5 was evaluated in different tissue and secondly, it was very small group of patients. Nevertheless, one can again speculate that the serum concentration of FABP5 may reflect its level in the skin. What was interesting, according to Miyake et al., skin- stripping FABP5 decreased prior to the clinical improvement, so it could help to predict the result of the treatment. In other study authors also observed decrease of FABP5 in psoriatic skin samples and serum after treatment with methotrexate [37]. Further prospective studies are needed to investigate if the serum concentration decrease also precedes the improvement of skin lesions after UVB. Reduction of FABP5 after treatment may indicate improvement of metabolic condition of psoriatic patients. Similar results (reduction of plasma FABP5) were obtained in morbid obese patients after gastric binding-induced weight loss [38]. Authors also suggested that FABP5 may be associated with metabolic improvement. In this context, it should further be noted that weight loss in obese psoriatic patients may improve skin lesions [39].

### Study strength and limitations

This study indicate not only higher concentration of serum FABP5 in psoriatic patients, in relationship with the severity of the disease and inflammatory markers but also the beneficial effect of treatment with NB-UVB on FABP5 concentration. The presented results point out two important informations for clinicians: first that psoriatic patients even without obesity have metabolic disturbances and should be screened for them, and second, even more important: that during our present study, after NB-UVB treatment we have observed decrease of serum FABP5 concentration which may have influence on metabolic status of psoriatic patients. The main limitation of the present study is lack of classic metabolic indices like TC, HDL-C, LDL-C after the NB-UVB. Observed significant reduction of PASI is strong evidence clinical improvement and reduction of FABP5 indicate improvement of metabolic status. It is difficult to verify if the observed changes in FABP5 levels are related to UV-therapy or to the improvement of psoriasis. Subsequent limitation of the study is small group of patients. To have more comprehensive view, it would be the best to extend the number of patients and make a comparison in subgroups with different comorbidities.

## Conclusion

In the present study elevated serum level of FABP5 in psoriatic patients correlates with the disease severity and accompanies indicators of systemic inflammation. These findings may suggest that serum concentration of FABP5 could be used as a novel clinical biomarker of inflammation and disease severity in psoriasis. Moreover, aberrant FABP5 indicate metabolic abnormalities which may result in the development of metabolic syndrome and cardiovascular diseases. The present results indicate the need and importance for screening of psoriatic patients in the direction of metabolic disturbances, even without associated obesity. Furthermore, decrease of FABP5 serum concentration during NB-UVB treatment may indicate the influence on metabolic status of psoriatic patients. This finding needs further investigations.

Clinicians, when deciding about psoriasis treatment should lean toward NB-UVB if it is possible, more often than topical treatment. Taken together, the results have additional value in the growing body of evidence indicating a common metabolic profile between psoriasis and cardiometabolic diseases. Therefore, understanding the possible relationship between them is an important issue that may lead to the development of more effective therapies.

## Data Availability

The datasets generated during and/or analysed during the current study are available from the corresponding author on reasonable request.

## References

[1] Gisondi P, Tessari G, Conti A, Piaserico S, Schianchi S, Peserico A, Giannetti A, Girolomoni A (2007). Prevalence of metabolic syndrome in patients with psoriasis: a hospital-based case-control study. Br J Dermatol.

[2] Singh S, Young P, Armstrong AW (2017). An update on psoriasis and metabolic syndrome: a meta-analysis of observational studies. PLoS ONE.

[3] Strand V, Gonçalves J, Hickling TP, Jones HJ, Marshall L, Isaacs JD (2020). Immunogenicity of biosimilars for rheumatic diseases, plaque psoriasis, and inflammatory bowel disease: a review from clinical trials and regulatory documents. BioDrugs.

[4] Miller IM, Ellervik C, Yazdanyar S, Jemec GBE (2013). Meta - analysis of psoriasis, cardiovascular disease, and associated risk factors. J Am Acad Dermatol.

[5] Magdaleno-Tapial J, Valenzuela-Oñate C, Ortiz-Salvador JM, Martínez-Doménech A, García-Legaz-Martínez M, Alonso-Carpio M, Tamarit-García JJ (2020). Prevalence of non-alcoholic fatty liver and liver fibrosis in patients with moderate-severe psoriasis: a cross-sectional cohort study. Australas J Dermatol.

[6] Chiricozzi A, Raimondo A, Lembo S, Fausti F, Dini V, Costanzo A, Monfrecola G (2016). Crosstalk between skin inflammation and adipose tissue-derived products: pathogenic evidence linking psoriasis to increased adiposity. Expert Rev Clin Immunol.

[7] Kamiya K, Kishimoto M, Sugai J, Komine M, Ohtsuki M (2019). Risk factors for the development of psoriasis. Int J Mol Sci.

[8] Mehta NN, Azfar RS, Shin DB, Neimann AL, Troxel AB, Gelfand JM (2010). Patients with severe psoriasis are at increased risk of cardiovascular mortality: cohort study using the general practice research database. Eur Heart J.

[9] Springate DA, Parisi R, Kontopantelis E, Reeves D, Griffiths CEM, Ashcroft DM (2017). Incidence, prevalence and mortality of patients with psoriasis: a U.K. population-based cohort study. Br J Dermatol.

[10] Furuhashi M, Hotamisligil GS (2008). Fatty acid-binding proteins: role in metabolic diseases and potential as drug targets. Nat Rev Drug Discov.

[11] Sikora M, Stec A, Chrabaszcz M, Waskiel-Burnat A, Zaremba M, Olszewska M, Rudnicka L (2019). Intestinal Fatty Acid Binding Protein, a biomarker of intestinal barrier is associated with severity of psoriasis. J Clin Med.

[12] Baran A, Świderska M, Bacharewicz-Szczerbicka J, Myśliwiec H, Flisiak I (2017). Serum fatty acid-binding protein 4 is increased in patients with psoriasis. Lipids.

[13] Khnykin D, Miner JH, Jahnsen F (2011). Role of fatty acid transporters in epidermis: implications for health and disease. Dermatoendocrinol.

[14] Smathers RL, Petersen DR (2011). The human fatty acid-binding protein family: evolutionary divergences and functions. Hum Genomics.

[15] Dallaglio K, Marconi A, Truzzi F, Lotti R, Palazzo E, Petrachi T, Saltari A (2013). E-FABP induces differentiation in normal human keratinocytes and modulates the differentiation process in psoriatic keratinocytes in vitro. Exp Dermatol.

[16] Bagheri R, Qasim AN, Mehta NN, Terembula K, Kapoor S, Braunstein S, Schutta M (2010). Relation of plasma fatty acid binding proteins 4 and 5 with the metabolic syndrome, inflammation and coronary calcium in patients with type-2 diabetes mellitus. Am J Cardiol.

[17] Schmitt J, Wozel G (2005). The Psoriasis area and severity index is the adequate criterion to define severity in chronic plaque-type psoriasis. Dermatology.

[18] Glaser C, Demmelmai H, Koletzko B (2010). High-throughput analysis of total plasma fatty acid composition with direct in situ transesterification. PLoS One.

[19] Miyake T, Ogawa E, Mikoshiba A, Kobayashi A, Hosoe H, Kashiwabara S, Uhara H (2012). Epidermal-type FABP is a predictive marker of clinical response to systemic treatment and ultraviolet therapy in psoriatic skin lesions. J Dermatol Sci.

[20] Masouye I, Saurat JH, Siegenthaler G (1996). Epidermal fatty-acid-binding protein in psoriasis, basal and squamous cell carcinomas: an immunohistological study. Dermatology.

[21] Furiati SC, Catarino JS, Silva MV, Silva RF, Estevam RB, Teodoro RB, Pereira SL, Ataide M (2019). Th1, Th17, and Treg responses are differently modulated by TNF- α inhibitors and methotrexate in psoriasis patients. Sci Rep.

[22] Li B, Reynolds JM, Stout RD, Bernlohr DA, Suttles J (2009). Regulation of Th17 differentiation by epidermal fatty acid-binding protein. J Immunol.

[23] Zhang Y, Li B (2014). E-FABP: regulator of immune function. Oncoscience.

[24] Rodriguez-Cerdeira C, Cordeiro-Rodriguez M, Carnero-Gregorio M, Lopez-Barcenas A, Martinez-Herrera E, Fabbrocini G (2019). Biomarkers of inflammation in obesity-psoriatic patients. Mediators Inflamm.

[25] Boehncke WH (2018). Systemic inflammation and cardiovascular comorbidity in psoriasis patients: causes and consequences. Front Immunol.

[26] Yeung DC, Wang Y, Xu A, Cheung SCW, Wat NMS, Fong DYT, Fong CHY (2008). Epidermal fatty-acid-binding protein: a new circulating biomarker associated with cardio-metabolic risk factors and carotid atherosclerosis. Eur Heart J.

[27] Ibarretxe D, Girona J, Amigó N, Plana N, Ferré R, Guaita S, Mallol R (2016). Impact of epidermal fatty acid binding protein on 2D-NMR-assessed atherogenic dyslipidemia and related disorders. J Clin Lipidol.

[28] Rodriguez-Zuniga MJM, Garcia-Perdomo HA (2017). Systematic review and meta-analysis of the association between psoriasis and metabolic syndrome. J Am Acad Dermatol.

[29] Puig L (2018). Cardiometabolic comorbidities in psoriasis and psoriatic arthritis. Int J Mol Sci.

[30] Setty AR, Curhan G, Choi HK (2007). Obesity, waist circumference, weight change, and the risk of psoriasis in women: nurses’ health study II. Arch Intern Med.

[31] Snekvik I, Smith CH, Nilsen TIL, Langan SM, Modalsli RPR, Saunes M (2017). Obesity, waist circumference, weight change, and risk of incident psoriasis: prospective data from the HUNT study. J Invest Dermatol.

[32] Snekvik I, Nilsen TIL, Romundstad PR, Saunes M (2019). Metabolic syndrome and risk of incident psoriasis: prospective data from the HUNT Study, Norway. Br J Dermatol.

[33] Zhang Y, Li Q, Rao E, Sun Y, Grossmann ME, Morris RJ, Cleary MP, Li B (2015). Epidermal fatty acid binding protein promotes skin inflammation induced by high-fat diet. Immunity.

[34] Sanson B, Wang T, Sun J, Wang L, Kaczocha M, Ojima I, Deutsch D, Li H (2014). Crystallographic study of FABP5 as an intracellular endocannabinoid transporter. Acta Crystallogr D Biol Crystallogr.

[35] Qin S, Wen J, Bai XC, Chen TY, Zheng RC, Zhou GB, Ma J (2014). Endogenous n-3 polyunsaturated fatty acids protect against imiquimod-induced psoriasis-like inflammation via the IL-17/IL-23 axis. Mol Med Rep.

[36] Ishimura S, Furuhashi M, Watanabe Y, Hoshina K, Fuseya T, Mita T, Okazaki Y (2013). Circulating levels of fatty acid-binding protein family and metabolic phenotype in the general population. PLoS ONE.

[37] Owczarczyk-Saczonek A, Czerwińska J, Orylska M, Placek W (2020). Effect of methotrexate treatment on the expression of epidermal fatty acid binding protein (E-FABP) and apolipoproteins in patients with psoriasis. Postepy Dermatol Alergol.

[38] Haider DG, Schindler K, Bohdjalian A, Prager G, Luger A, Wolzt M, Ludvik B (2007). Plasma adipocyte and epidermal fatty acid binding protein is reduced after weight loss in obesity. Diabetes Obes Metab.

[39] Alotaibi HA (2018). Effect of weight loss on psoriasis: a review of clinical trials. Cureus.

